# Plasmid-Mediated Quinolone Resistance (PMQR) in Two Clinical Strains of *Salmonella enterica* Serovar Corvallis

**DOI:** 10.3390/microorganisms10030579

**Published:** 2022-03-07

**Authors:** Xenia Vázquez, Javier Fernández, Silvia Hernáez, Rosaura Rodicio, Maria Rosario Rodicio

**Affiliations:** 1 Departamento de Biología Funcional, Área de Microbiología, Universidad de Oviedo (UO), 33006 Oviedo, Spain; xenia_grao@hotmail.com; 2 Grupo de Microbiología Traslacional, Instituto de Investigación Sanitaria del Principado de Asturias (ISPA), 33011 Oviedo, Spain; javifdom@gmail.com (J.F.); mrosaura@uniovi.es (R.R.); 3 Servicio de Microbiología, Hospital Universitario Central de Asturias (HUCA), 33011 Oviedo, Spain; 4 Research & Innovation, Artificial Intelligence and Statistical Department, Pragmatech AI Solutions, 33003 Oviedo, Spain; 5 Centro de Investigación Biomédica en Red-Enfermedades Respiratorias, 28029 Madrid, Spain; 6 Servicio de Microbiología, Hospital Universitario de Álava (HUA), 01009 Álava, Spain; silvia.hernaezcrespo@osakidetza.eus; 7 Departamento de Bioquímica y Biología Molecular, Universidad de Oviedo (UO), 33003 Oviedo, Spain

**Keywords:** *Salmonella enterica* serovar Corvallis, ST1541, PMQR, *qnrS1*, ColE, IncQ1, whole genome sequencing, phylogenetic analysis

## Abstract

Non-typhoid serovars of *Salmonella enterica* are one of the main causes of bacterial food-borne infections worldwide. For the treatment of severe cases of salmonellosis in adults, fluoroquinolones are amongst the drugs of choice. They are categorized by the World Health Organization (WHO) as “critically important with highest priority in human medicine”. In the present study, two clinical *S. enterica* serovar Corvallis isolates (HUA 5/18 and HUA 6/18) from a Spanish hospital, selected on the basis of fluoroquinolone resistance, were characterized. The MICs of ciprofloxacin, determined by E-test, were 0.5 and 0.75 µg/mL for HUA 5/18 and HUA 6/18, respectively, and both were also resistant to pefloxacin but susceptible to nalidixic acid. Whole genome sequencing (WGS) of the isolates was performed with Illumina platform, and different bioinformatics tools were used for sequence analysis. The two isolates belonged to ST1541, and had the Thr57Ser substitution in the ParC protein which is also found in ciprofloxacin susceptible isolates. However, they harbored identical ColE plasmids of 10 kb carrying the *qnrS1* gene. In these plasmids, the gene was flanked by defective versions of IS*2*-like and IS*Kra4*-like insertion sequences. HUA 5/18 and HUA 6/18 were also phenotypically resistant to streptomycin, sulfonamides and tetracycline, with the responsible genes: *strA*, *strB*, *sul2* and *tet*(A) genes, being located on a IncQ1 plasmid. ColE plasmids with the *qnrS1* gene are widely spread among multiple serovars of *S. enterica* from different samples and countries. These mobilizable plasmids are playing an important role in the worldwide spread of *qnrS1*. Thus, their detection in hospitals is a cause of concern which deserves further attention.

## 1. Introduction

*Salmonella enterica* subsp. *enterica* serovar Corvallis, with antigenic formula 8,20:z4,z23, is a cause of food-borne disease in humans, which has been mainly linked to consumption of poultry meat and derived products. According to the last report of the European Union (EU) on zoonoses, this serovar ranked around the 40th position from 2017 to 2019 [1]. However, a higher incidence has been observed in particular countries, like Bulgaria, Denmark, Thailand, Japan and Brazil [2,3,4,5]. In addition, *S*. Corvallis isolates resistant to third generation cephalosporins, carbapenems, colistin and fluoroquinolones have been reported [2,3,5,6,7]. All these antimicrobials are listed by the World Health Organization (WHO) as “critically important for human medicine”, because of their application in the effective control of serious bacterial infections [8].

Fluoroquinolones are among the drugs of choice for the treatment of severe cases of salmonellosis in adults, and *S. enterica* resistant to these antimicrobials is amongst the high priority pathogens listed by WHO [9]. Fluoroquinolone resistance in *S. enterica* has been mainly associated with point mutations affecting the quinolone resistance determinant region (QRDR) of chromosomal genes encoding the subunits of DNA topoisomerase II (DNA gyrase; *gyrA* and *gyrB*) and DNA topoisomerase IV (*parC* and *parD*), which are involved in DNA replication and transcription. In Gram-negative bacteria, mutations usually occur in a sequential way, and have an additive effect. Thus, a point mutation affecting the QRDR of *gyrA* and leading to nalidixic acid resistance can be followed by additional mutations in *gyrA* and/or other target genes like *parC*, which, combined with the former, extends resistance to fluoroquinolones. Other chromosomal mutations, leading to decreased expression of outer membrane porins or to overexpression of multidrug efflux pumps, can also have an impact on susceptibility to quinolones by reducing the intracellular drug accumulation [10,11,12,13,14]. More recently, plasmid mediated quinolone resistance (PMQR) was detected, raising concerns about transferability of this important resistance. In *Enterobacteriaceae*, PMQR is conferred by *qnr* (*qnrA, qnrB, qnrC*, *qnrD*, *qnrS* and *qnrVC*), *qep*, *oqxAB* or *aac(6′)-Ib-cr* genes. The Qnr proteins, which are members of the pentapeptide-repeat family, protect DNA gyrase and DNA topoisomerase IV from fluoroquinolone inhibition; QepA and OqxAB are efflux pumps of the major facilitator superfamily (MFS) and the resistance-nodulation division family (RND), respectively, involved in the active expulsion of fluoroquinolones; and Aac(6′)-Ib-cr is a bifunctional acetyltransferase, capable of modifying both aminoglycosides and quinolones [15,16]. Although PMQR genes usually confer only low-level resistance to fluoroquinolones without concomitant resistance to quinolones, they provide a genetic background for selection of chromosomal mechanisms leading to higher resistance levels, apart from impairing treatment [13].

In the present study we investigated the genetic bases of quinolone and fluoroquinolone resistance in two clinical isolates of *S. enterica* serovar Corvallis detected in a Spanish hospital. For this, experimental approaches were combined with whole genome sequencing (WGS) followed by bioinformatics analysis.

## 2. Materials and Methods

### 2.1. Bacterial Isolates and Antimicrobial Susceptibility

Two *S*. Corvallis isolates, HUA 5/18 and HUA 6/18, selected for their ciprofloxacin resistance, were analyzed in the present study. They were recovered at the “Hospital Universitario de Álava” (HUA), Basque Country, Spain, from fecal samples of different patients, both with acute gastroenteritis. The bacteria were isolated on selective culture media (Selenite broth and Hecktoen agar; bioMerieux, Marcy l’Etoile, France) and identified by MALDI-TOF (Bruker Daltonics, Billerica, MA), according to the manufacturer’s instructions. Briefly, a small amount of inoculum from fresh colonies of the isolates was smeared on a polished steel MSP 96 target plate (Bruker Daltonik), overlaid with 1 μL of a saturated alfa-cyano-4-hydroxy-cinnamic acid (alfa-CHCA) matrix solution in 50% acetonitrile-2.5% trifluoroacetic acid (Bruker Daltonik), and air dried at room temperature. The spectra were then acquired by the mass spectrometer and compared with those included in the reference database using the BioTyper software. Susceptibility to antimicrobial agents was determined by automated MicroScan NC 53 (Beckman Coulter, CA, USA), complemented with Bauer–Kirby disk diffusion assays, using commercially available discs (Oxoid, Madrid, Spain). The following compounds, with the amount per disk in µg shown in parenthesis, were used: ampicillin (10), amoxicillin-clavulanic acid (30), cefepime (30), cefotaxime (30), cefoxitin (30), erthapenem (10), chloramphenicol (30), amikacin (30), gentamicin (10), kanamycin (30), streptomycin (10), tobramycin (10), azithromycin (15), nalidixic acid (30), ciprofloxacin (5), sulfonamides (300), tetracycline (30), trimethoprim (5), fosfomycin (300) and nitrofurantoin (300). MICs to nalidixic acid and ciprofloxacin were determined by E-test (bioMérieux, Marcy l’Étoile, France). In accordance with the EUCAST (European Committee on Antimicrobial Susceptibility Testing) guidelines (https://eucast.org/clinical_breakpoints/ (accessed on 4 November 2021)), a 5 µg pefloxacin disk was included in the Bauer–Kirby assays, as surrogate to detect clinical resistance to fluoroquinolones. The reason for this is that the nalidixic acid disk used (30 µg) fails to effectively detect isolates with PMQR, and because of the overlap in the inhibition zone diameter (IZD) between wild-type isolates and isolates with low-level resistance observed for the 5 µg ciprofloxacin disk. Results were interpreted according to EUCAST or to CLSI (Clinical and Laboratory Standards Institute) guidelines [17], the latter in the case of NAL that is not contemplated by EUCAST.

### 2.2. Whole Genome Sequencing, Bioinformatics Analysis and Phylogenetic Relationships

Genomic DNA was purified from overnight cultures grown in Luria–Bertani (LB) broth, with the GenElute^TM^ Bacterial Genomic DNA Kit (Sigma-Aldrich; Merck Life Science, Madrid, Spain), following the manufacturer’s instructions. WGS was performed with Illumina at the “Centro de Investigación Biomédica”, La Rioja (CIBIR), Spain, to generate paired-end reads of 100–150 nt from PCR-free fragment libraries of 400–500 bp. *De novo* assembly of the reads was accomplished with the VelvetOptimiser.pl script implemented in the “on line” version of PLACNETw (https://omictools.com/placnet-tool/ (accessed on 6 June 2019)), which also allowed separating contigs of chromosomal and plasmid origin [18]. Relevant information related to the quality of the assemblies is shown in Table 1.

The assembled genomes of the isolates, deposited in GenBank under the accession numbers included in “Data Availability Statement” (see below), were annotated with the NCBI Prokaryotic Genome Annotation Pipeline (PGAP; https://www.ncbi.nlm.nih.gov/genome/annotation_prok/ (accessed on 17 May 2021)). For bioinformatics analysis, PLACNETw and different tools available at the Center for Genomic Epidemiology (CGE) of the Technical University of Denmark (DTU) were used (https://cge.cbs.dtu.dk/services/ (accessed on 8 November 2021)). These included MLST (v2.0.4), ResFinder (v4.1) and PlasmidFinder (v2.0.1) [19,20,21,22]. The phylogenetic relationships between the *S*. Corvallis isolates from HUA and 44 *S*. Corvallis isolates retrieved from databases were inferred using the CSI phylogeny tool (v1.4), available at the CGE website [23]. The pipeline was run with default parameters, using the genome of HUA 6/18 as the reference for SNP calling. Bootstrap support for the consensus phylogenetic tree relied on 1000 replicates [24]. The accession numbers of the genomes included in the phylogenetic analysis and the resulting SNP matrix are shown in Tables S1 and S2, respectively. The annotations of the resistance plasmids carrying either the *qnrS1* gene or other resistance genes were manually curated with the aid of blastn, blastp (https://blast.ncbi.nlm.nih.gov/Blast.cgi (accessed on 25 November 2021)) and CLONE Manager Professional (CmSuit9). The genetic organization of these plasmids was represented with EasyFig BLASTn (https://mjsull.github.io/Easyfig/ (accessed on 6 December 2021)).

## 3. Results and Discussion

HUA 5/18 and HUA 6/18 were recovered from feces of a 53 year-old female and a 6 year-old child with acute gastroenteritis, attended at a hospital in Northern Spain (Table 2). They were selected on the basis of resistance to fluoroquinolones (see below), and experimentally assigned to *S. enterica* serogroup C2 at the hospital. The assembly size of the sequenced genomes was of 4.89 Mb for both isolates (Table 1), with a GC content of approximately 52.1%. Serotyping and MLST performed in silico identified the isolates as *S*. Corvallis of sequence type ST1541. Five small plasmids, with sizes ranging from 2.2 to 11 kb, were shared by HUA 5/18 and HUA 6/18. They belonged to the IncQ1, ColE and ColpVC incompatibility groups, or had an unidentified replicon.

The two isolates were susceptible to nalidixic acid, with IZD of 20 mm in disk diffusion assays, and MICs of 3 and 4 µg/mL for HUA 5/18 and HUA 6/18, respectively. In contrast, the MICs of ciprofloxacin were 0.5 and 0.75 µg/mL for HUA 5/18 and HUA 6/18, respectively, well above the breakpoint of resistance established by EUCAST. In agreement with this, both were resistant to pefloxacin with an IZD of 12 mm around the 5 µg disk used in the Bauer–Kirby assay recommended by EUCAST to detect clinical resistance to fluoroquinolones.

**Figure 1:**
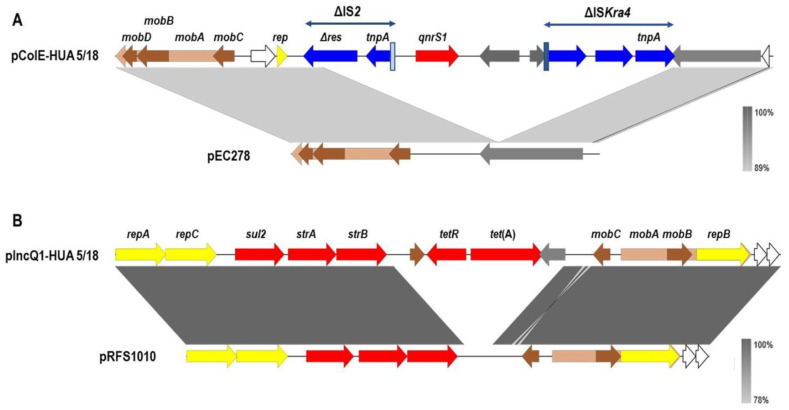
Genetic organization of the ColE (**A**) and IncQ1 (**B**) plasmids carried by *Salmonella enterica* serovar Corvallis isolates from a Spanish hospital. The alignments of the ColE and IncQ1 plasmids with pEC278 (accession number AY589571) and pRFS1010 (accession number M28829), respectively, were created with EasyFig BLASTn. The gray shading between regions reflects nucleotide sequence identities according to the scales shown at the right lower corner of the figures. The open reading frames (ORFs) are represented by arrows pointing to the direction of transcription and having different colors according to their function: red, resistance; yellow, plasmid replication; brown, mobilization; blue, insertion sequences; grey, other roles; white, hypothetical proteins. Vertical boxes indicate truncated (pale blue) and intact (dark blue) inverted repeats of IS*2*-like and ISKar4-like elements, respectively. The *mobA* gene in the ColE plasmids (pale brown) partially (*mobC*) or totally (*mobB* and *mobD*) overlaps the other *mob* genes. The *mobA* gene of the IncQ plasmids (pale brown) overlaps with *mobB* and *repB*.

As revealed by bioinformatics analysis, HUA 5/18 and HUA 6/18 contained the PMQR gene *qnrS1*, which is likely to account for the low-level ciprofloxacin resistance shown by the isolates. This gene has been previously found in clinical, veterinary and food-borne isolates of different *S. enterica* serovars, including *S*. Corvallis [6,25,26,27,28,29,30]. In *S. enterica*, *qnrS1* has been located on plasmids belonging to different incompatibility groups, comprising IncN, IncHI2, IncR, IncX and ColE [26,27,31,32,33,34,35]. The isolates in this study carried the gene on ColE plasmids which were identical to each other and nearly identical (˃99.5%) to other fully sequenced plasmids found in clinical isolates of *S*. Typhimurium from the UK and Taiwan [36,37,38], and to plasmids of *S*. Corvallis and *S*. Typhimurium recovered in Japan from seafood imported from Thailand and Vietnam [25]. Like these other plasmids, they contain the *mobA*, *mobB*, *mobC* and *mobD* genes coding for mobilization proteins similar to those expressed by the *E. coli* pEC278 plasmid (accession number AY589571), and a *rep* gene which is more closely related to that of the *E. coli* ColRNA1 plasmid p15A (accession number V00309; [39]), (Figure 1A). Although ColE-like plasmids are not self-transmissible, they can be mobilized by co-resident conjugative plasmids due to the presence of *oriT* and *mob* genes [36]. Several environments have been reported for the *qnrS1* gene, which was found to be associated with different mobile genetic elements, including Tn*3*-like transposons and insertion sequences IS*Ecl*, IS*26* and IS*2*-like [15,37]. The *qnrS1* gene in the ColE plasmids analyzed herein, was preceded by two *orfs* which share the highest homology with *orfs* of the IS*2*-like elements IS*As17* and IS*Ecl1*, and it was followed by a truncated IS*Kpn19* element of the IS*Kra4* family (Figure 1A).

None of the two isolates under study carried mutations in the QRDR of *gyrA*, *gyrB* and *parE*. However, both had AGC at codon 57 of *parC*, instead of ACC that is usually found in *S. enterica*. This change, which results in Thr57Ser substitution in the ParC protein, was previously found in quinolone-susceptible isolates of serovars *S*. Schwarzengrund, *S*. Hadar and *S*. Kentucky [40,41,42], as well as in a quinolone-susceptible isolate of *S*. Corvallis found in seafood imported in USA from Thailand [25] and in three quinolone-susceptible clinical isolates of the same serovar tested in our laboratory (unpublished results). These results argue against a role of the Thr57Ser substitution in resistance. Moreover, the genomes of 44 S. Corvallis isolates retrieved from databases to perform a phylogenetic analysis in the present study (see below), also had Ser instead of Thr as the 57th amino acid in ParC, although information on quinolone susceptibility of the latter isolates was not always available. Ser instead of Thr57 was also observed to be unrelated to quinolone resistance in the ParC protein of *Shigella flexneri* and of several *E. coli* serovars, indicating not only a cross-serovar but also a cross-species distribution of this polymorphism [43].

In addition to ciprofloxacin, HUA 5/18 and HUA 6/18 were also resistant to streptomycin, sulfonamides and tetracycline (Table 2). The responsible genes were identified as *strA*, *strB*, *sul2* and *tet*(A), harbored by a IncQ1 plasmid of about 11 kb, which was identical in the two isolates (Figure 1B). Closely related plasmids were reported in several strains of *S*. Typhimurium recovered from humans, food-producing animals and foods in different countries, including Italy, UK and Taiwan (accession numbers CP004059, KU852461 and CP025338, for plasmids pNUC, pSTU288-2 and p11k, respectively) [44,45,46], and also in single strains of three other serovars, namely *S*. Reading, *S*. Schwarzengrund and *S*. Corvallis (accession numbers CP082735, CP081859 and CP044201, for unpublished plasmids pN16S0842, p16 an pAR-046-1, respectively). All these plasmids displayed more than 99.5% identity, with 100% coverage. In addition, the plasmids of HUA 5/18 and HUA 6/18 shared 99% identity with 74% coverage with pRFS1010 (of 8,684 bp), a multi-copy broad-host range plasmid of *Escherichia coli*, which is non-conjugative but can be mobilized by co-resident conjugative plasmids (accession number M28829); [47]. pRFS1010 is one of the most extensively studied IncQ1 plasmids, and is considered as a prototype for the IncQ group [48]. The homologous region comprises genes involved in replication (*repA*, *repB* and *repC*), and mobilization (*mobA*, *mobB* and *mobC*), and also the *strA*, *strB* and *sul2* genes. In contrast, the *tetR* and *tet*(A) genes are absent in pRFS1010, but present in the *S*. Corvallis and closely related plasmids (Figure 1B). It has been proposed that the latter plasmids constitute a separate group of IncQ plasmids, which have evolved from pRFS1010 through acquisition of the *tetR*-*tet*(A) genes, characteristically carried by transposon Tn*1721* [46]. As previously noticed for the homologous pSTU288-2 of *S*. Typhimurium [45], the GC content of the *S*. Corvallis plasmid (61.7%) is well above the GC content of the host strains (52.1%). Like in HUD 5/18 and HUD 6/18, both the ColE and the IncQ plasmids were found together in five closely related strains of *S*. Typhimurium obtained from humans and animals in Taiwan [44].

**Figure 2:**
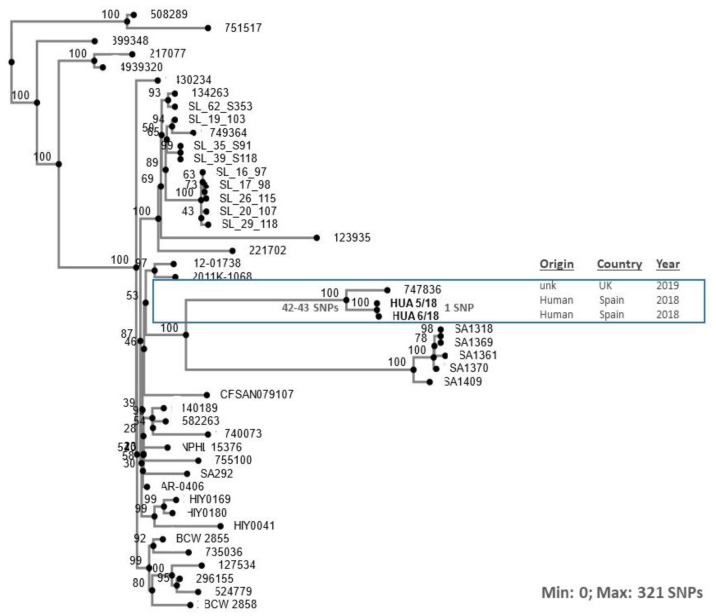
Phylogenetic tree showing the relationships between *Salmonella enterica* serovar Corvallis ST1541 isolates from a Spanish hospital and other *S*. Corvallis ST1541 isolates from different countries and sources (Table S1). The minimum and maximum numbers of SNPs are indicated (see Table S2). The branch containing the Spanish isolates (highlighted in bold) is enclosed in a rectangle, which includes information available for the isolates shown at the right of the figure (unk = unknown). The tree was constructed with the CSI Phylogeny, based on single nucleotide polymorphisms identified by using the genome of *S*. Corvallis HUA 6/18 as reference. Numbers at the nodes represent bootstrap values resulting from 1000 replicates.

Finally, a phylogenetic tree was constructed with the two newly sequenced genomes of *S*. Corvallis and 44 other genomes available in public databases (Table S1), all with sequence type ST1541 which is the most commonly associated with this serovar. As shown in Figure 2, the clinical isolates from the Basque Country, which were nearly identical to each other, belong to a branch that also contains a single isolate from the UK (accession number AAFGXP010000001.1) whose origin has not been described, but which is also positive for the *qnrS1* gene according to ResFinder. The two isolates in the present study were recovered from different patients with no travel history before the onset of the disease. They were obtained at about the same time, and could have been associated with a small outbreak, although information on a possible epidemiological link between the two patients was not available. In any case, it is interesting that closely related *qnrS1*-positive isolates, which differ by only 42–43 SNPs are circulating in two different countries, i.e., Spain and UK, indicating the international spread of such isolates.

## 4. Conclusions

Two clinical isolates of *S*. Corvallis with low-level resistance to ciprofloxacin: HUA 5/18 and HUA 6/18, carried the Thr57Ser substitution in the ParC protein, and a ColE1 plasmid bearing the *qnrS1* gene. The Thr57Ser substitution in ParC was also found in susceptible isolates. Accordingly, the PMQR *qnrS1* gene is likely to be responsible for the fluoroquinolone resistance phenotype of HUA 5/18 and HUA 6/18. Nearly identical *qnrS1* positive isolates are circulating in different countries, consistent with their international spread.

## Figures and Tables

**Table 1:** Parameters related to the quality of the assemblies of the genomes of two clinical isolates of *Salmonella enterica* serovar Corvallis resistant to fluoroquinolones.

Isolate ^a^	Kmer	Contigs	N50	Longest Contig (bp)	Total bp in Contigs	Contigs >1 kb	Library	Coverage
HUA 5/18	85	78	795,230	2,081,448	4,887,704	22	508 ± 126	25×
HUA 6/18	87	131	407,429	1,548,594	4,886,051	46	506 ± 129	41×

^a^, Isolates are designated with the initials of the center which supplied them, followed by a serial number/last two numbers of the year of recovery. HUA, “Hospital Universitario de Álava”, Basque Country, Spain.

**Table 2:** Origin, resistance properties and plasmid content of *Salmonella enterica* serovar Corvallis ST1541.

Isolate ^a^	Patient Sex ^b^/Age	NAL-IZD ^c^ (mm)	NAL-MIC ^c^ (µg/mL)	CIP-MIC ^c^ (µg/mL)	PEF-IZD ^c^ (mm)	ParC	Resistance Phenotype ^d^ Plasmid-Located Resistance Genes	Plasmid Inc. (Size in bp) ^e^
HUA 5/18	F/53	20	3	0.5	12	Thr57Ser	CIP, PEF, STR, SUL, TET *strA, strB, sul2, tet*(A) *qnrS1*	**IncQ1** (11,044)* **ColE** (10,036)* nid (5,570; 5,284)*,* ColpVC (2,179)
HUA 6/18	F/6	20	4	0.75	12	Thr57Ser	STR, SUL, TET, CIP, PEF *strA, strB, sul2, tet*(A) *qnrS1*	**IncQ1** (11,044)* **ColE** (10,036)* nid (5,570; 5,284)*,* ColpVC (2,179)

^a^, HUA, “Hospital Universitario de Álava”, Basque Country, Spain. ^b^, F, female. ^c^, NAL, nalidixic acid; CIP, ciprofloxacin; PEF, pefloxacin; IZD, inhibition zone diameter; MIC, minimum inhibitory concentration. ^d^, STR, streptomycin; SUL, sulfonamides; TET, tetracycline. ^e^, Inc, incompatibility group, with resistance plasmids shown in bold; nid, Inc not identified; *, circularized plasmids.

## Data Availability

The genome sequences generated in the present study were deposited in GenBank under accession numbers JAHCQI000000000 and JAHCQJ000000000 for *Salmonella enterica* serovar Corvallis HUA 5/18 and HUA 6/18, respectively.

## Supplementary Materials

The following are available online at https://www.mdpi.com/article/10.3390/microorganisms10030579/s1, Table S1: Accession numbers of the genomes and additional information on the *Salmonella enterica* serovar Corvallis ST1541 isolates used for phylogenetic analysis in the present study. Table S2: Pairwise SNP distance matrix calculated from the genomes of two *Salmonella enterica* serovar Corvallis human isolates from a Spanish hospital and 44 isolates from other sources and countries.
