# Supplementary material for: Plasmid-Mediated Quinolone Resistance (PMQR) in Two Clinical Strains of Salmonella enterica Serovar Corvallis
Source: Microorganisms. 2022 Mar 7;10(3):579. doi: 10.3390/microorganisms10030579 (PMC8953408; doi:10.3390/microorganisms10030579)
Supplement: Supplementary file 1 [file microorganisms-10-00579-s001.zip › microorganisms-1557440-supplementary.pdf]

microorganisms

Supplementary Materials

Table S1. Accession numbers of the genomes and additional information on the *Salmonella enterica* serovar Corvallis ST1541 isolates used for phylogenetic analysis in the present study.

Isolate	Genome size (bp)	Origin	Country	Year of isolation	GenBank/ENA accession no.	Reference
12-01738	4,887,378	Wild bird	Germany	2012	CP027677.1	[1]
AR-0406	4,799,356	ukn	-	-	CP044200.1	unpub
2011K-1068	4,804,015	ukn	USA	-	MYYS01000001.1	unpub
127534	4,816,168	ukn	United Kingdom	2015	RTAB01000001.1	unpub
221702	4,752,286	ukn	United Kingdom	2016	RSSF01000001.1	unpub
399348	4,898,914	ukn	United Kingdom	2017	RTNL01000001.1	unpub
BCW_2855	4,719,825	ukn	Denmark	-	MXXG01000001.1	unpub
BCW_2858	5,020,070	ukn	Denmark	-	MXXD01000001.1	unpub
582263	4,767,427	ukn	United Kingdom	2018	RURT01000001.1	unpub
217077	5,015,564	ukn	United Kingdom	2016	RUXP01000001.1	unpub
140189	4,757,520	ukn	United Kingdom	2015	AAABOE010000001.1	unpub
134263	4,842,163	ukn	United Kingdom	2015	AAABSC010000001.1	unpub
524779	4,814,503	ukn	United Kingdom	2018	AAACAJ010000001.1	unpub
430234	4,784,350	ukn	United Kingdom	2017	AAACCW010000001.1	unpub
296155	4,816,897	ukn	United Kingdom	2016	AAACFW010000001.1	unpub
123935	5,040,541	ukn	United Kingdom	2015	AAAGCH010000001.1	unpub
SL_62_S353	4,899,668	Human stool	Sri Lanka	2016	SMQT01000001.1	[2]
SL_39_S118	4,860,207	Human blood	Sri Lanka	2016	SMPY01000001.1	[2]
SL_35_S91	4,861,639	Human stool	Sri Lanka	2017	SMPU01000001.1	[2]
CFSAN079107	4,872,954	Kratom Capsules	USA	2018	CP042440	[3]
SL_29_118	4,868,926	Raw chicken meat	Sri Lanka	2015	SMPP01000001.1	[2]
SL_26_115	4,990,834	Raw chicken meat	Sri Lanka	2015	SMPM01000001.1	[2]
SL_20_107	4,991,033	Raw chicken meat	Sri Lanka	2015	SMPG01000001.1	[2]
SL_17_98	4,984,047	Raw chicken meat	Sri Lanka	2015	SMPD01000001.1	[2]
SL_16_97	4,992,136	Raw chicken meat	Sri Lanka	2015	SMPC01000001.1	[2]
SL_19_103	4,902,599	Raw chicken meat	Sri Lanka	2015	SMPF01000001.1	[2]
HIY0169	4,764,495	Municipal wastewater, Sand Island WWTP	USA	2010	VCVD02000001.1	unpub
747836	4,961,984	ukn	United Kingdom	2019	AAFGXP010000001.1	unpub
751517	5,236,478	ukn	United Kingdom	2019	AAFHFR010000001.1	unpub
749364	4,898,242	ukn	United Kingdom	2019	AAFIFA010000001.1	unpub
740073	4,909,507	ukn	United Kingdom	2019	AAFIJW010000001.1	unpub
755100	4,893,052	ukn	United Kingdom	2019	AAFIKN010000001.1	unpub
735036	4,777,186	ukn	United Kingdom	2019	AAFJBU010000001.1	unpub
HIY0180	4,765,527	Municipal wastewater, Sand Island WWTP	USA	2010	VLRF01000001.1	unpub
HIY0041	4,684,888	Municipal wastewater, Sand Island WWTP	USA	2010	VLSM01000001.1	unpub
508289	5,056,816	ukn	United Kingdom	2018	AAIJDD010000001.1	unpub

NPHL 15376	5,013,778	Human blood	USA	2016	SPQE01000001.1	[4]
SA1409	4,953,145	Beef from supermarket	China	2016	JAEKPE010000001.1	[5]
SA1370	4,954,294	Chicken from wild market	China	2016	JAEKPW010000001.1	[5]
SA1369	4,958,591	Chicken from wild market	China	2016	JAEKPX010000001.1	[5]
SA1361	5,020,005	Chicken from supermarket	China	2016	JAEKQB010000001.1	[5]
SA1318	4,960,826	Chicken from wild market	China	2016	JAEKQV010000001.1	[5]
SA292	5,015,091	Chicken from wild market	China	2015	JAELMQ010000001.1	[5]
SA1249	4,939,320	Chicken from wild market	China	2016	JAEKSN010000001.1	[5]

ukn, unknown.

Table S2. Pairwise SNP distance matrix calculated from the genomes of two *Salmonella enterica* serovar Corvallis human isolates from a Spanish hospital and 44 isolates from other sources and countries.

	12-01738	123935	127534	134263	140189	2011K-1068	217077	221702	296155	399348	430234	4939320	508289	524779	582263	735036	740073	747836	749364	751517	755100	AR-0406	BCW_2855	BCW_2858	CFSAN079107	HIY0041	HIY0169	HIY0180	HUA 5/18	HUA 6/18	NPHL_15376	SA1318	SA1361	SA1369	SA1370	SA1409	SA292	SL_16_97	SL_17_98	SL_19_103	SL_20_107	SL_26_115	SL_29_118	SL_35_S91	SL_39_S118	SL_62_S353
12-01738		113	58	39	31	23	102	70	46	105	35	87	149	56	32	51	55	146	50	184	50	22	37	52	52	62	38	36	140	141	33	169	171	169	167	164	44	54	56	39	56	56	57	42	42	39
123935	113		133	94	108	114	177	127	121	180	110	162	224	131	109	126	132	223	105	259	127	99	112	127	131	139	115	113	217	218	110	248	250	248	246	243	121	109	111	94	111	111	112	97	97	94
127534	58	133		59	53	59	120	90	22	123	49	105	167	32	54	53	77	170	70	202	72	44	39	50	76	84	60	58	162	163	55	193	195	193	191	188	66	74	76	59	76	76	77	62	62	59
134263	39	94	59		34	40	103	51	47	106	36	88	150	57	35	52	58	149	27	185	53	25	38	53	57	65	41	39	143	144	36	174	176	174	172	169	47	31	33	16	33	33	34	19	19	8
140189	31	108	53	34		32	97	65	41	100	30	82	144	51	15	46	38	143	45	179	43	15	32	47	49	57	33	31	137	138	26	166	168	166	164	161	37	49	51	34	51	51	52	37	37	34
2011K-1068	23	114	59	40	32		103	71	47	106	36	88	150	57	33	52	56	147	51	185	51	23	38	53	53	63	39	37	141	142	34	170	172	170	168	165	45	55	57	40	57	57	58	43	43	40
217077	102	177	120	103	97	103		134	108	87	97	27	131	118	98	113	121	212	114	166	116	88	99	114	120	128	104	102	206	207	99	237	239	237	235	232	110	118	120	103	120	120	121	106	106	103
221702	70	127	90	51	65	71	134		78	137	67	119	181	88	66	83	89	180	62	216	84	56	69	84	88	94	72	70	174	175	67	205	207	205	203	200	78	66	68	51	68	68	69	54	54	51
296155	46	121	22	47	41	47	108	78		111	37	93	155	14	42	41	65	158	58	190	60	32	27	38	64	72	48	46	152	153	43	181	183	181	179	176	54	62	64	47	64	64	65	50	50	47
399348	105	180	123	106	100	106	87	137	111		100	72	114	121	101	116	124	215	117	149	119	91	102	117	123	131	107	105	209	210	102	240	242	240	238	235	113	121	123	106	123	123	124	109	109	106
430234	35	110	49	36	30	36	97	67	37	100		82	144	47	31	42	54	147	47	179	49	21	28	43	53	61	37	35	141	142	32	170	172	170	168	165	43	51	53	36	53	53	54	39	39	36
4939320	87	162	105	88	82	88	27	119	93	72	82		116	103	83	98	106	197	99	151	101	73	84	99	105	113	89	87	191	192	84	222	224	222	220	217	95	103	105	88	105	105	106	91	91	88
508289	149	224	167	150	144	150	131	181	155	114	144	116		165	145	160	168	259	161	51	163	135	146	161	167	175	151	149	253	254	146	284	286	284	282	279	157	165	167	150	167	167	168	153	153	150
524779	56	131	32	57	51	57	118	88	14	121	47	103	165		52	51	75	168	68	200	70	42	37	48	74	82	58	56	162	163	53	191	193	191	189	186	64	72	74	57	74	74	75	60	60	57
582263	32	109	54	35	15	33	98	66	42	101	31	83	145	52		47	39	144	46	180	44	16	33	48	50	58	34	32	138	139	27	167	169	167	165	162	38	50	52	35	52	52	53	38	38	35
735036	51	126	53	52	46	52	113	83	41	116	42	98	160	51	47		70	163	63	195	65	37	26	45	69	77	53	51	157	158	48	186	188	186	184	181	59	67	69	52	69	69	70	55	55	52
740073	55	132	77	58	38	56	121	89	65	124	54	106	168	75	39	70		167	69	203	67	39	56	71	73	81	57	55	161	162	50	190	192	190	188	185	61	73	75	58	75	75	76	61	61	58
747836	146	223	170	149	143	147	212	180	158	215	147	197	259	168	144	163	167		160	294	162	134	147	164	164	174	150	148	42	43	145	231	233	231	229	226	152	164	166	149	166	166	167	152	152	149
749364	50	105	70	27	45	51	114	62	58	117	47	99	161	68	46	63	69	160		196	64	36	49	64	68	76	52	50	154	155	47	185	187	185	183	180	58	38	40	15	40	40	41	24	24	27
751517	184	259	202	185	179	185	166	216	190	149	179	151	51	200	180	195	203	294	196		198	170	181	196	202	210	186	184	288	289	181	319	321	319	317	314	192	200	202	185	202	202	203	188	188	185
755100	50	127	72	53	43	51	116	84	60	119	49	101	163	70	44	65	67	162	64	198		34	51	66	68	76	52	50	156	157	45	185	187	185	183	180	56	68	70	53	70	70	71	56	56	53
AR-0406	22	99	44	25	15	23	88	56	32	91	21	73	135	42	16	37	39	134	36	170	34		23	38	40	48	24	22	128	129	17	157	159	157	155	152	28	40	42	25	42	42	43	28	28	25
BCW_2855	37	112	39	38	32	38	99	69	27	102	28	84	146	37	33	26	56	147	49	181	51	23		31	55	63	39	37	141	142	34	172	174	172	170	167	45	53	55	38	55	55	56	41	41	38
BCW_2858	52	127	50	53	47	53	114	84	38	117	43	99	161	48	48	45	71	164	64	196	66	38	31		70	78	54	52	158	159	49	187	189	187	185	182	60	68	70	53	70	70	71	56	56	53
CFSAN079107	52	131	76	57	49	53	120	88	64	123	53	105	167	74	50	69	73	164	68	202	68	40	55	70		80	56	54	158	159	51	187	189	187	185	182	62	72	74	57	74	74	75	60	60	57
HIY0041	62	139	84	65	57	63	128	94	72	131	61	113	175	82	58	77	81	174	76	210	76	48	63	78	80		50	48	168	169	59	197	199	197	195	192	70	80	82	65	82	82	83	68	68	65
HIY0169	38	115	60	41	33	39	104	72	48	107	37	89	151	58	34	53	57	150	52	186	52	24	39	54	56	50		12	144	145	35	173	175	173	171	168	46	56	58	41	58	58	59	44	44	41
HIY0180	36	113	58	39	31	37	102	70	46	105	35	87	149	56	32	51	55	148	50	184	50	22	37	52	54	48	12		142	143	33	171	173	171	169	166	44	54	56	39	56	56	57	42	42	39
HUA 5/18	140	217	162	143	137	141	206	174	152	209	141	191	253	162	138	157	161	42	154	288	156	128	141	158	158	168	144	142		1	139	225	227	225	223	220	146	158	160	143	160	160	161	146	146	143
HUA 6/18	141	218	163	144	138	142	207	175	153	210	142	192	254	163	139	158	162	43	155	289	157	129	142	159	159	169	145	143	1		140	226	228	226	224	221	147	159	161	144	161	161	162	147	147	144
NPHL_15376	33	110	55	36	26	34	99	67	43	102	32	84	146	53	27	48	50	145	47	181	45	17	34	49	51	59	35	33	139	140		168	170	168	166	163	39	51	53	36	53	53	54	39	39	36
SA1318	169	248	193	174	166	170	237	205	181	240	170	222	284	191	167	186	190	231	185	319	185	157	172	187	187	197	173	171	225	226	168		10	0	6	25	179	189	191	174	191	191	192	177	177	174
SA1361	171	250	195	176	168	172	239	207	183	242	172	224	286	193	169	188	192	233	187	321	187	159	174	189	189	199	175	173	227	228	170	10		10	8	27	181	191	193	176	193	193	194	179	179	176
SA1369	169	248	193	174	166	170	237	205	181	240	170	222	284	191	167	186	190	231	185	319	185	157	172	187	187	197	173	171	225	226	168	0	10		6	25	179	189	191	174	191	191	192	177	177	174
SA1370	167	246	191	172	164	168	235	203	179	238	168	220	282	189	165	184	188	229	183	317	183	155	170	185	185	195	171	169	223	224	166	6	8	6		23	177	187	189	172	189	189	190	175	175	172
SA1409	164	243	188	169	161	165	232	200	176	235	165	217	279	186	162	181	185	226	180	314	180	152	167	182	182	192	168	166	220	221	163	25	27	25	23		174	184	186	169	186	186	187	172	172	169
SA292	44	121	66	47	37	45	110	78	54	113	43	95	157	64	38	59	61	152	58	192	56	28	45	60	62	70	46	44	146	147	39	179	181	179	177	174		62	64	47	64	64	65	50	50	47
SL_16_97	54	109	74	31	49	55	118	66	62	121	51	103	165	72	50	67	73	164	38	200	68	40	53	68	72	80	56	54	158	159	51	189	191	189	187	184	62		2	27	4	2	5	30	30	31
SL_17_98	56	111	76	33	51	57	120	68	64	123	53	105	167	74	52	69	75	166	40	202	70	42	55	70	74	82	58	56	160	161	53	191	193	191	189	186	64	2		29	6	2	7	32	32	33
SL_19_103	39	94	59	16	34	40	103	51	47	106	36	88	150	57	35	52	58	149	15	185	53	25	38	53	57	65	41	39	143	144	36	174	176	174	172	169	47	27	29		29	29	30	13	13	16
SL_20_107	56	111	76	33	51	57	120	68	64	123	53	105	167	74	52	69	75	166	40	202	70	42	55	70	74	82	58	56	160	161	53	191	193	191	189	186	64	4	6	29		6	7	32	32	33
SL_26_115	56	111	76	33	51	57	120	68	64	123	53	105	167	74	52	69	75	166	40	202	70	42	55	70	74	82	58	56	160	161	53	191	193	191	189	186	64	2	2	29	6		7	32	32	33
SL_29_118	57	112	77	34	52	58	121	69	65	124	54	106	168	75	53	70	76	167	41	203	71	43	56	71	75	83	59	57	161	162	54	192	194	192	190	187	65	5	7	30	7	7		33	33	34
SL_35_S91	42	97	62	19	37	43	106	54	50	109	39	91	153	60	38	55	61	152	24	188	56	28	41	56	60	68	44	42	146	147	39	177	179	177	175	172	50	30	32	13	32	32	33		0	19
SL_39_S118	42	97	62	19	37	43	106	54	50	109	39	91	153	60	38	55	61	152	24	188	56	28	41	56	60	68	44	42	146	147	39	177	179	177	175	172	50	30	32	13	32	32	33	0		19
SL_62_S353	39	94	59	8	34	40	103	51	47	106	36	88	150	57	35	52	58	149	27	185	53	25	38	53	57	65	41	39	143	144	36	174	176	174	172	169	47	31	33	16	33	33	34	19	19	
